# Acute inhibition of hunger-sensing AgRP neurons promotes context-specific learning in mice

**DOI:** 10.1016/j.molmet.2023.101803

**Published:** 2023-09-09

**Authors:** Felicia Reed, Alex Reichenbach, Harry Dempsey, Rachel E. Clarke, Mathieu Mequinion, Romana Stark, Sasha Rawlinson, Claire J. Foldi, Sarah H. Lockie, Zane B. Andrews

**Affiliations:** 1 Monash Biomedicine Discovery Institute and Department of Physiology, Monash University, Clayton, 3800, Victoria, Australia; 2 Department of Neurosciences, Medical University of South Carolina, Charleston, SC, 29425, USA

**Keywords:** AgRP neurons, Photometry, Optogenetics, Chemogenetics, Hunger, Conditioning

## Abstract

**Objective:**

An environmental context, which reliably predicts food availability, can increase the appetitive food drive within the same environment context. However, hunger is required for the development of such a context-induced feeding (CIF) response, suggesting the neural circuits sensitive to hunger link an internal energy state with a particular environment context. Since Agouti related peptide (AgRP) neurons are activated by energy deficit, we hypothesised that AgRP neurons are both necessary and sufficient to drive CIF.

**Methods:**

To examine the role of AgRP neurons in the CIF process, we used fibre photometry with GCaMP7f, chemogenetic activation of AgRP neurons, as well as optogenetic control of AgRP neurons to facilitate acute temporal control not permitted with chemogenetics.

**Results:**

A CIF response at test was only observed when mice were fasted during context training and AgRP population activity at test showed an attenuated inhibitory response to food, suggesting increased food-seeking and/or decreased satiety signalling drives the increased feeding response at test. Intriguingly, chemogenetic activation of AgRP neurons during context training did not increase CIF, suggesting precise temporal firing properties may be required. Indeed, termination of AgRP neuronal photostimulation during context training (ON–OFF in context), in the presence or absence of food, increased CIF. Moreover, photoinhibition of AgRP neurons during context training in fasted mice was sufficient to drive a subsequent CIF in the absence of food.

**Conclusions:**

Our results suggest that AgRP neurons regulate the acquisition of CIF when the acute inhibition of AgRP activity is temporally matched to context exposure. These results establish acute AgRP inhibition as a salient neural event underscoring the effect of hunger on associative learning.

## Highlights

• Fasted mice learn to associate an environmental context with calorie consumption, which increases subsequent food intake in the same context in the absence of hunger.
• This context-induced feeding response depends on dynamic changes in AgRP neuronal activity.
• Optogenetic photostimulation and cessation of AgRP neurons drives context-induced feeding, even when calorie consumption is absent during training.
• Photoinhibition of fasting-induced AgRP activity drives context-induced feeding, despite no food being consumed during training.
• Chemogenetic excitation of AgRP neurons does not induce context-induced feeding, as it overrides temporal AgRP inhibitory responses to food.
• AgRP neurons regulate the acquisition of context-induced feeding when AgRP neuronal inhibition is matched to context exposure.

## 1 Introduction

Hunger acts as a powerful incentive for an animal to interact resourcefully with its environment, and many higher-order cognitive processes likely evolved to support reliable food-finding under conditions of energy deficit. Hunger elicited by food restriction is frequently used as a motivational tool to accelerate acquisition of appetitive learning tasks in the laboratory [[1], [2], [3]], despite limited knowledge of how hunger affects information processing in the brain. Hunger-associated conditions such as calorie restriction (CR), time-restricted feeding, or exogenous administration of ghrelin improve acquisition in simple object recognition tasks [4,5]. Not only can hunger promote memory, but this link appears to be reciprocal as memories of past experiences influence current levels of appetite. For example, situations previously paired with hunger can elicit conditioned feeding responses in the absence of energy deficit [[6], [7], [8]], while situations signalling aversive outcomes can suppress appetite and food intake, despite energy need [9]. Although a large number of studies demonstrate the effect of appetite on memory, the neurobiological processes and neural circuits linking appetite and memory remain poorly understood.

Hunger is associated with increased activity of AgRP neurons located in the arcuate nucleus of the hypothalamus [[10], [11], [12]]. Chemogenetic and optogenetic activation of these neurons is sufficient to drive feeding and appetitive behaviours comparable to those elicited by fasting [13,14]. Activation of these neurons in fed mice induces recall of a location-specific memory acquired under food restriction [15] or a specific operant behavioural sequence previously rewarded under food restricted conditions [11]. In addition, AgRP neuronal activation influences mood [[16], [17], [18]], dopamine driven motivation [19], reward, reinforcement, valence attribution and associative learning [[20], [21], [22], [23], [24]].

Under physiological conditions, AgRP neurons are silenced prior to food consumption in response to sensory cues that predict food [10,19,21,25]. The fall in activity depends on both the current energy need of the animal and the calorie content of available food, with the greatest suppression occurring in fasted mice or when mice are presented with palatable food or calorie-containing gels [25,26]. A sustained decrease in AgRP activity requires both metabolic sensing in AgRP neurons [19] and gastrointestinal feedback via increased gut peptide signalling and gut-brain neural feedback [[26], [27], [28]], which act to confirm calorie intake and content. For example, when mice are re-exposed to novel flavoured food gels, a sustained suppression of AgRP neuronal activity only occurs in response to consuming caloric gels compared to non-caloric gels; clearly demonstrating the necessity of nutrient dependent feedback in this process [26]. Moreover, inactivation of an afferent inhibitory GABAergic dorsomedial hypothalamic (DMH) pathway to AgRP neurons delays learning in a visual cue-initiated food-seeking task [23]. Thus, the acute inhibitory control of AgRP neurons seems important to guide learning. Based on these findings, we have recently argued that the sensory and temporal integration of food or food cues regulate AgRP neurons in a predictive and experience-dependent manner [29].

Collectively, the evidence presented above suggests that AgRP neurons provide a critical bidirectional link between hunger and memory. Given that AgRP neurons sense interoceptive hunger states [11] and hunger elicits conditioned feeding responses paired with distinct contextual information [[6], [7], [8],^30^], we hypothesised that AgRP neurons encode interoceptive- and context-dependent information into conditioned feeding responses. Moreover, the rapid inhibition of AgRP neurons in response to the sensory detection of food suggests that the temporal silencing of AgRP neurons themselves is critical to facilitate conditioning to environmental cues. Therefore, we assessed the specific temporal role of AgRP neuronal activity in response to environmental cues using a context-induced feeding assay, independent of energy need.

## 2 Methods

### 2.1 Mice and housing

All experiments were conducted in accordance with the Monash Animal Ethics Committee guidelines, (MARP 18012). Mice were group housed under standard conditions (12:12 light–dark cycle, lights off at 7 pm) and given *ad libitum* access to standard chow diet (no. 8720610, Barastoc Stockfeeds, Victoria, Australia) prior to surgical intervention.

*Agrp-ires-cre* mice on a C57BL/6J background (*Agrp*^*tm1(cre)Low/J*^) were obtained from Jackson Laboratory (stock no. 012899; The Jackson Laboratory, Maine, USA) and crossed with C57BL/6J mice obtained from the Monash Animal Research Platform (MARP; Clayton, AU). Male offspring heterozygous or wild-type (wt) for the cre allele were used for experiments (*Agrp*^cre/wt^ and *Agrp*^wt/wt^; respectively). Following surgeries, all mice were single housed and given free access to Irradiated Rat and Mouse Maintenance Chow (4.8% fat, 19% protein, 60% carbohydrates; Specialty Feeds, Australia) for a minimum of 2 weeks prior to experimentation.

Adult male C57Bl/6J mice aged 12 weeks on arrival were obtained from MARP and group housed 2–4 mice per cage under the same standard conditions and allowed to acclimate to the behavioural facility for a minimum of 7 days before experimentation.

### 2.2 Stereotaxic surgery

Stereotaxic surgeries were performed on adult male *Agrp*^cre/wt^ and *Agrp*^wt/wt^ littermates at least 10 weeks of age. For all surgeries, *Agrp*^cre/wt^ and *Agrp*^wt/wt^ mice received bilateral injections of AAV (∼2.0 × 10^12^ vg/ml), 200 nl/side infused at a rate of 40 nl/min and allowed to rest for 5 min post-infusion. All viruses were purchased from Addgene (AAV5-hSyn-DIO-hM3D(Gq)-mCherry, #50474; AAV9 hSynapsin-FLEX-soCoChR-GFP, #107712; AAV1-hSyn-SIO-stGtACR2-FusionRed, #105677; AAV9-syn-FLEX-jGCaMP7f-WPRE, #104492) and injected into the mid-arcuate nucleus using a 2 μl Hamilton Neuros Syringe (Stereotaxic co-ordinates, from bregma: AP: −1.7 mm, ML: ±0.2 mm, DV: −5.8 mm from brain surface). For optogenetic experiments, mice additionally received a unilateral fibre optic implant into the left hemisphere with 5.8 mm long ferrule capped fibres (400 μm core, NA 0.48, MF1.25 or MF 2.5 400/430–0.48; Doric studios, Canada) above the injection site and fixed in place with G-flow dental adhesive (GBond, GC Dental, Japan). Mice had a minimum of 2 weeks post-surgical recovery time, also permitting time for viral transduction.

### 2.3 Drugs and compounds

Clozapine-N-oxide (CNO) (Carbosynth, UK) was dissolved in sterile saline (0.9% NaCl) for injections. For Designer Receptor Exclusively Activated by Designer Drug (DREADD) activation studies (hM3Dq), a concentration of 1 mg/kg was used. CNO was injected intraperitoneally (i.p) at a volume of 10 μl/g of body weight.

Purified rat ghrelin (R&D Systems, USA) was dissolved in saline on the day of injection. Experimental mice received a dose of 1 mg/kg, which significantly increased food intake, delivered i.p. at a volume of 10 μl/g of body weight.

### 2.4 AgRP photometry

AgRP neuronal population activity was measured through the excitation and emission of GCaMP7f expressed in AgRP neurons using optical components from Doric Lenses (Quebec, Canada) controlled by Tucker Davis Technologies (TDT) processor (RZ10x). A calcium-dependent GCaMP7f signal was detected using light at 465 nm, whereas a 405 nm wavelength served as a calcium-independent isosbestic control for artifactual movement. Signal demodulation and data were acquired using TDT Synapse software and customized Python Code was used for data analysis (openly available on GitHub: https://github.com/Andrews-Lab/Fiber_photometry_analysis). The fluorescence of 405 nm was subtracted from the fluorescence of 465 nm using the equation: $\frac{df}{f}=\left(f465\;\text{nm}-f405\;\text{nm}\right)/f405\;\text{nm}$ to account for background fluorescence, minimize photobleaching and any calcium-independent fluorescence such as motions artifact. Behavioural events of interest were timestamped and precisely aligned to the neuronal data using the TDT OpenScope software. Z-score normalisation was used to allow for standard comparisons between mice according to the following equation: $Z=\left(x-x_{o}\right)/S$, where x denotes raw data points, while $x_{o}$ and S represent the mean and standard deviation of defined baseline.

### 2.5 Optogenetics

Optogenetic experiments were conducted using optical components from Doric Lenses (Quebec, Canada). 10 mW of 465 nm blue light was delivered from an LED driver to a connectorized LED coupled with a fibre optic rotary joint. Light output power was standardized to 10 mW per setup using an Optical Power Meter (PM100D, ThorLabs, NJ USA) measured at the end of the fibre optic patch cable. For CIF and home cage feeding experiments, the following photostimulation parameters were used: 20 Hz pulses delivered every 1 in 4 s (1 s ON, 3 s OFF), based on the reported firing frequency of AgRP neurons during fasting *in vivo* [10]. For photoinhibition, 10 mW of 465 nm blue light was delivered constantly. Pulse generation was controlled by an optogenetics TTL pulse generator using Doric Neuroscience Studio software.

### 2.6 Functional validation of AgRP neuron activation with optogenetics or DREADDs

Chemogenetic or optogenetic stimulation of AgRP neurons drives robust food intake in fed mice [13,14], thus we used home cage food intake as a functional readout of successful expression of hM3Dq DREADDs or soma-targeted channelrhodopsin (soCoChR2) expression in AgRP neurons. For hM3Dq DREADDs experiments, mice were administered CNO (1 mg/kg) before food access was reinstated 1 h later. Food and body weight were then recorded at 2 h- and 5 h-post re-feeding. Mice that showed an elevated food intake response at 2 h were deemed eligible for inclusion in the experiment. For optogenetic experiments, we used the food intake values reported during the 30 min of stimulation given during the training period to prevent any pretraining conditioning effects of transferring mice to the testing room. This time frame (10 min home-cage pre-simulation + 20 min within-context stimulation) was sufficient to drive a significant increase in feeding behaviour in *Agrp*^cre/wt^ but not *Agrp*^wt/wt^ mice. To examine how optogenetic inhibition affected home cage feeding, AgRP^gtACR2^ and AgRP^WT^ mice were fasted for 3 h. Home cage food intake was measured for 30 min during the early dark phase with photoinhibition during the refeeding period.

### 2.7 Immunohistochemistry

Successful viral transfection was confirmed by the presence of reporter expression in the Arc of *Agrp*^cre/wt^ and the absence from *Agrp*^wt/wt^ (mCherry: AAV5-hSyn-DIO-hM3D(Gq)-mCherry; or GFP: AAV9 hSynapsin-FLEX-SoCoChR-GFP), which was detected by immunohistochemistry. Following behavioural experiments, mice were transcardially perfused with 0.05 M phosphate buffered saline (PBS) followed by 4% paraformaldehyde (PFA) in PBS. Following overnight post-fixation in PFA, brains were transferred to a 30% sucrose in 0.1 M PB solution for a minimum of 24 h prior to cryosectioning. Sections were cut on a cryostat at 35 μm and stored in cryoprotectant at −20 °C prior to processing. Free-floating sections were washed in 0.1 M phosphate buffer (PB) followed by blocking in 4% normal horse serum (NHS) in 0.1 M PB + 0.3% Triton-X. Primary antibodies were added to the blocking solution (Rabbit anti-DsRed 1:1000, stock #632496, Takara Bio, Clonetech; chicken anti-GFP 1:1000, ab13970, Abcam) and incubated overnight at 4 °C. The next day, sections were washed in 0.1 M PB before secondary antibody incubation for 2 h at room temperature (Alexafluor Goat anti-rabbit 594 or Goat anti-chicken 488; 1:400 in 0.1 M PB). Sections were mounted onto a slide and coverslipped using Vectorshield antifade mounting medium with DAPI (Vector Laboratories, Newark, CA USA).

### 2.8 Context-induced feeding (CIF) assay

The CIF assay is a rapid, validated task in which food consumption is linked by association with an environmental context. Once paired, this association drives food consumption in sated mice. It provides a framework for studying the underlying neurobiology of feeding in the absence of current hunger [7].

#### 2.8.1 Habituation

Prior to training, age-matched mice were habituated to palatable Froot Loops® breakfast cereal (168 kJ/g; 84.3% carbohydrate, 5.4% protein, 3.6% fat, Kellogg's, Australia) in their home cage on 3 separate occasions at different times of day. On the day prior to training, mice were also habituated to 2 separate contexts for 20 min each, in the absence of any food. Order of exposure to the contexts was counterbalanced across treatment groups. The contexts were designed to be distinct sensory experiences, differing in location (room); colour, shape, and texture of the testing arena (round yellow washing tub with a smooth floor surface, vs a comparably sized grey rectangular storage crate with an inserted ribbed flooring); smell (one of the following essences – rosewater, vanilla, rum, or almond); as well as placement and type of dish used as a food receptacle (ceramic dish vs 50 ml falcon tube lid or petri dish). All animals were habituated to handling and received a minimum of 3 daily i.p. injections prior to experimentation, or 3 days habituation to tethered cables for optogenetic studies.

#### 2.8.2 Training/acquisition of context-feeding

Training sessions involved placing mice into one of the contexts (assigned context A) for 30 min each, repeated across 3 days. Training in context A was paired with roughly 600–800 mg of a chopped mixture of Froot Loops placed inside the food dish. All contexts were wiped down with warm water and 50ul of appropriate olfactory cue reintroduced for each training session. All mice were trained within a 4-h window in the mid-light phase, with food removed 1 h prior to training (to eliminate the effects of recent feeding bouts) and returned 1 h after they were returned to their home cage. This was repeated across 3 consecutive days for all experiments.

#### 2.8.3 Testing/recall of context-induced feeding

Mice were re-fed in their home cages and tested across 2 days, 24–48 h following the final training session. The presence of a conditioned feeding response was evaluated by comparing 20-min palatable food intake in context A (the training context) to 20-min intake in context B (a familiar, but untrained context). Order of context exposure was counterbalanced across days, and tests in each context were performed at the same time of day to prevent any time-of-day variation in feeding. All testing was performed in the absence of any treatment to assess the effect of treatment on acquisition.

### 2.9 CIF with fed, fasted, and ghrelin-treated C57Bl/6J mice

Experiments were performed as outlined above with the following exceptions. C57Bl/6J mice were randomly assigned to the following groups for training: Fasted + saline (FASTED; n = 9), Fed + saline (FED; n = 10), and Fed + 1 mg/kg ghrelin (GHRELIN; n = 10). Prior to each of the training days, mice allocated to the FASTED group had no food access starting from 2 h before light onset, for a total of 10–12 h before each training day. Following training, mice were re-fed 1 h following training. For the FED- and GHRELIN-treated groups, food was removed concurrent with i.p. injection, which was performed 30 min prior to training. Access to home-cage food was returned to all mice 1 h after the training session.

### 2.10 AgRP neural population activity during training and CIF

Mice expressing jGCaMP7f in AgRP neurons were habituated to context A and B, as above. During training, 10–12 h fasted mice were connected to patch cords for 10 min prior to starting recordings. Mice were recorded in home cages for 10 min before being transferred into context A, with Froot Loops presented 10 min later (Figure 2B). On the test day, fed mice were connected to patch cords and AgRP activity was recorded for 10 min prior to transfer into either context A or context B. During the testing of CIF, Froot Loops were added after 10 min to enable a clear distinction in recordings before and after the addition of Froot Loops.

### 2.11 CIF in fasted mice with Gq DREADD activation

Experiments were performed as outlined above with the following exceptions. AgRP^WT^ and AgRP^hM3Dq^ mice were trained under *ad libitum* (fed) conditions during the mid-light phase. Food was removed and all mice received i.p. injections of CNO (1 mg/kg) 1 h prior to being placed into the context. All mice had access to Froot Loops in the training context. ***Test:*** all mice were tested for 20 min in each context under fed conditions, with the same food present in context A or B.

### 2.12 CIF in fed mice with AgRP photostimulation or photoinhibition

#### 2.12.1 Experiment 1 – photostimulation with food

Experiments were performed as outlined above with the following exceptions. Fed mice were tethered to fibre optic cables and allowed to settle for 10 min in their home cage prior to photostimulation. Photostimulation commenced 10 min prior to transfer in the training context and training sessions were 30 min in duration, which included an initial 20 min of photostimulation (20 Hz, 1 s ON, 3 s OFF) followed by 10 min within the context but in the absence of photostimulation. This was intended to drive an acute feeding response in fed mice, with an additional period following food consumption during which AgRP neurons were not photostimulated. Testing was performed without stimulation.

#### 2.12.2 Experiment 2 – photostimulation without food in context

Experiments were performed as outlined above with the following exceptions. Fed mice were transferred to the training room and received 10 min of photostimulation in the home cage (20 Hz, 1 s ON, 3 s OFF) before transfer into the training context. Photostimulation was terminated 10 min into the 30-min training session, which was performed in the absence of any food in the training context. The cessation of AgRP photostimulation within the training context was designed to mirror the silencing of AgRP neurons that naturally occurs in response to food presentation, to assess whether conditioning could be achieved in the absence of food and post-ingestive feedback during training. The presence of CIF was evaluated in the absence of photostimulation on the test day.

#### 2.12.3 Experiment 3 – constant photostimulation without food

Fed mice were transferred to the training room and received 10 min of photostimulation in the home cage (20 Hz, 1 s ON, 3 s OFF) before transfer into the training context. Within the training context, mice received AgRP photostimulation for the entire 30-min training session in the absence of any food in the training context. Mice were transferred back to their home cages and the cessation of AgRP photostimulation occurred 10 min later. This approach prevented any change in AgRP activity from being associated with the training context. The presence of CIF was evaluated in the absence of photostimulation on the test day.

#### 2.12.4 Experiment 4 – context-specific photoinhibition of AgRP neurons in fasted mice

Mice were fasted for 6 h prior to training sessions with all training sessions occurring ∼2 h into the dark phase, when mice normally consume the most food. After been transferred to the training room and tether to optical cables, mice were placed in the training context for 10 min prior to starting AgRP photoinhibition (constant 10 mW of 465 nm blue light). No food was available in the training context. Mice were transferred back to their home cages and photoinhibition was terminated 5 min later to avoid any change in AgRP activity to be associated with the training context. The presence of CIF was evaluated in the absence of photoinhibition on the test day.

### 2.13 Video analysis of behaviour

CIF experiments were recorded for video analysis of fed, fasted and ghrelin-treated C57Bl/6J mice using a fixed overhead camera (Sony HDR-50 Action Cam). Videos were scored using Ethovision tracking software (Noldus, US) to determine frequency, duration (s) and latency (s) for each pre-defined zone, as well as total session activity expressed as velocity (cm/s) and distance travelled (cm). For the CIF experiments, the zones included the food zone (immediately surrounding the food receptacle), as well as a ‘food approach’ zone extending an approx. 5 cm radius outside the food zone, and the remaining floor zone. Where described, behavioural barcoding across the training and test was performed on the videos using a combination of manual scoring and an in-house python analysis script, to establish the breakdown in behaviours in each zone over time.

### 2.14 Statistical information

Data were analysed using Prism 9 for MacOS (GraphPad Software, USA). Comparisons involving 2 factors (e.g. context, genotype) were analysed by 2-way ANOVA and Tukey's post hoc test for multiple comparisons. Experiments involving within-animal comparisons were analysed using 2-way RM ANOVA followed by Sidak's post hoc test for multiple comparisons. Tests comparing genotype alone for a single time point were performed using an unpaired t-test. Significance for all tests was reported at the level of p < 0.05. All statistical information is reported in Supplemental Table 1.

## 3 Results

### 3.1 Mice trained under fasted, but not fed or ghrelin-treated conditions acquire context-induced feeding

**Figure 1:**
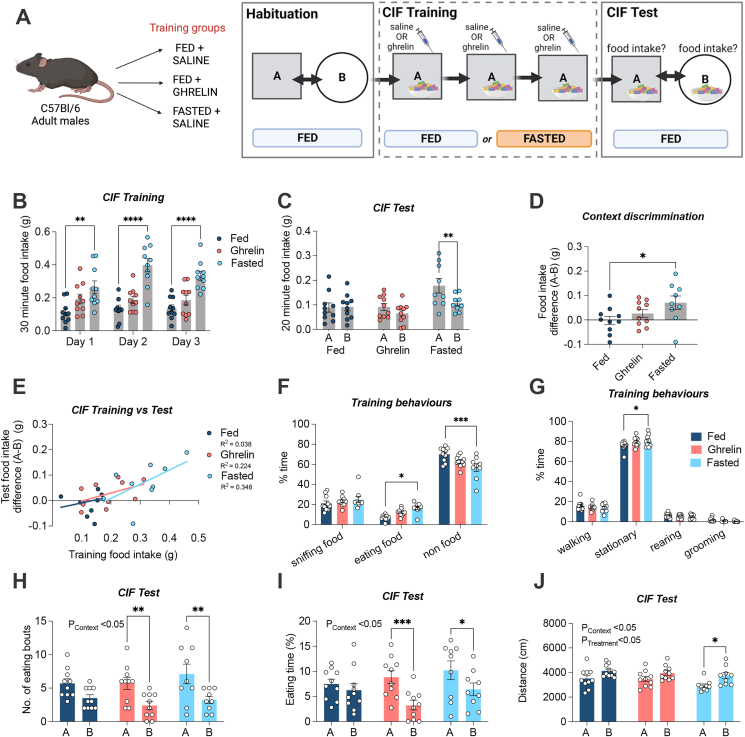
**Context induced feeding (CIF) in C57Bl/6J mice trained under fed, ghrelin-treated or fasted conditions.** (A) Adult C57Bl/6J mice were split into 3 groups for CIF training: fed + saline (i.p) (n = 10), fed + ghrelin (1 mg/kg, i.p) (n = 10), and fasted + saline (i.p) (n = 9). Following habituation to contexts A and B, all mice received 3 days of training in context A paired with palatable Froot Loops and were then tested for a CIF response by comparing intake in context A to B; schematic created with BioRender.com. (B) CIF training food intake for fed, ghrelin-treated and fasted groups across each of the 30-min training sessions. (C) The presence of a CIF response at test was evaluated in *ad libitum* fed mice by comparing food intake in context A to B during 20-min test sessions. (D) CIF test data expressed as the difference in food intake in context A and B (A–B; one-way ANOVA with Dunnett's post hoc test for multiple comparisons). (E) Relationship between food consumed during training and context discrimination at test. (F) Breakdown of % time engaged in food- and non-food related behaviours during training in in all 3 groups. (G) Breakdown of % time engaging in specific non-food behaviours during training (walking, stationary, rearing, grooming) in all 3 groups. (H) Number of eating bouts during CIF testing in A and B; (I) % time spent eating during CIF testing in A and B and (J) Locomotor activity (total distance travelled) during testing in context A and B for all 3 groups. All values are expressed as mean ± SEM. Data analysed by 2-way RM ANOVA with Sidak's post hoc test for multiple comparisons, unless otherwise stated. ∗p < 0.05, ∗∗p < 0.01, ∗∗∗p < 0.001, ∗∗∗∗p < 0.0001. All specific statistical information is reported in Supplemental Table 1.

We first examined the effects of fasting and ghrelin treatment, given both ghrelin and fasting are known to act on AgRP neurons to drive feeding (Figure 1A). Compared to Fed controls, Fasted mice ate significantly greater amounts across each of the training sessions (p < 0.05; Figure 1B). Ghrelin-treated mice trended to eat mice on average across the 3 training days, although this was not significant in the overall comparison, with a trend toward increased intake evident only on the first day (p = 0.073). When all mice were re-fed and later tested for CIF, only the Fasted group displayed a CIF response based on food intake in context A vs context B (p = 0.007; Figure 1C) or when context discrimination was expressed as the individual difference in food intake between context A vs context B (p = 0.034; Figure 1D). Fasted mice also showed the strongest relationship between average training food intake and context discrimination for the CIF test (R^2^ = 0.348), although this was not significant (A–B; Figure 1E).

We also profiled the behaviours of mice from Fed, Ghrelin-treated and Fasted groups on the first and last training days (Day 1 and Day 3) and the two test days (Contexts A and B), focussing on the first 10 min of each trial when changes in appetitive behaviour are likely to be most prominent. Fasted mice exhibited the greatest food-oriented behaviour during training; spending significantly more time eating food (p = 0.01; Figure 1F), and an overall reduction in time engaging in non-food related behaviours (p value; Figure 1F). Ghrelin-treated mice showed an intermediate response between Fed and Fasted mice for both behaviours although this was not significantly different from Fed mice. When we separately characterised non-food related behaviours, all groups spent most of their time stationary, but this was only significantly higher in the Fasted group due to the fact they spent more time eating food (p = 0.030; Figure 1G). There were no differences in time spent sniffing food (Figure 1F), walking, rearing, or grooming (Figures 1G).

When food intake in context A was compared to B in the CIF test, mice trained in the Fasted and Ghrelin groups engaged in more bouts of feeding (0.001; Figure 1H) and the Ghrelin group spent significantly more time eating (p = 0.008; Figure 1I), whereas mice trained in the Fed group displayed similar feeding behaviour in both contexts. Overall mice displayed greater locomotor activity in context B compared to A (p = 0.005), but a significant difference between contexts was only apparent in the Fasted group (p = 0.029; Figure 1J). Altogether, these results are consistent with Fasted mice engaging in more food-oriented behaviours during training, which led to the formation of food-specific conditioned feeding behaviour at test. Although the Ghrelin group displayed some behavioural differences at test, our results suggest the 3-day conditioning approach with ghrelin was insufficient to elicit a CIF response at test.

### 3.2 Context-specific AgRP neuronal population activity during CIF

**Figure 2:**
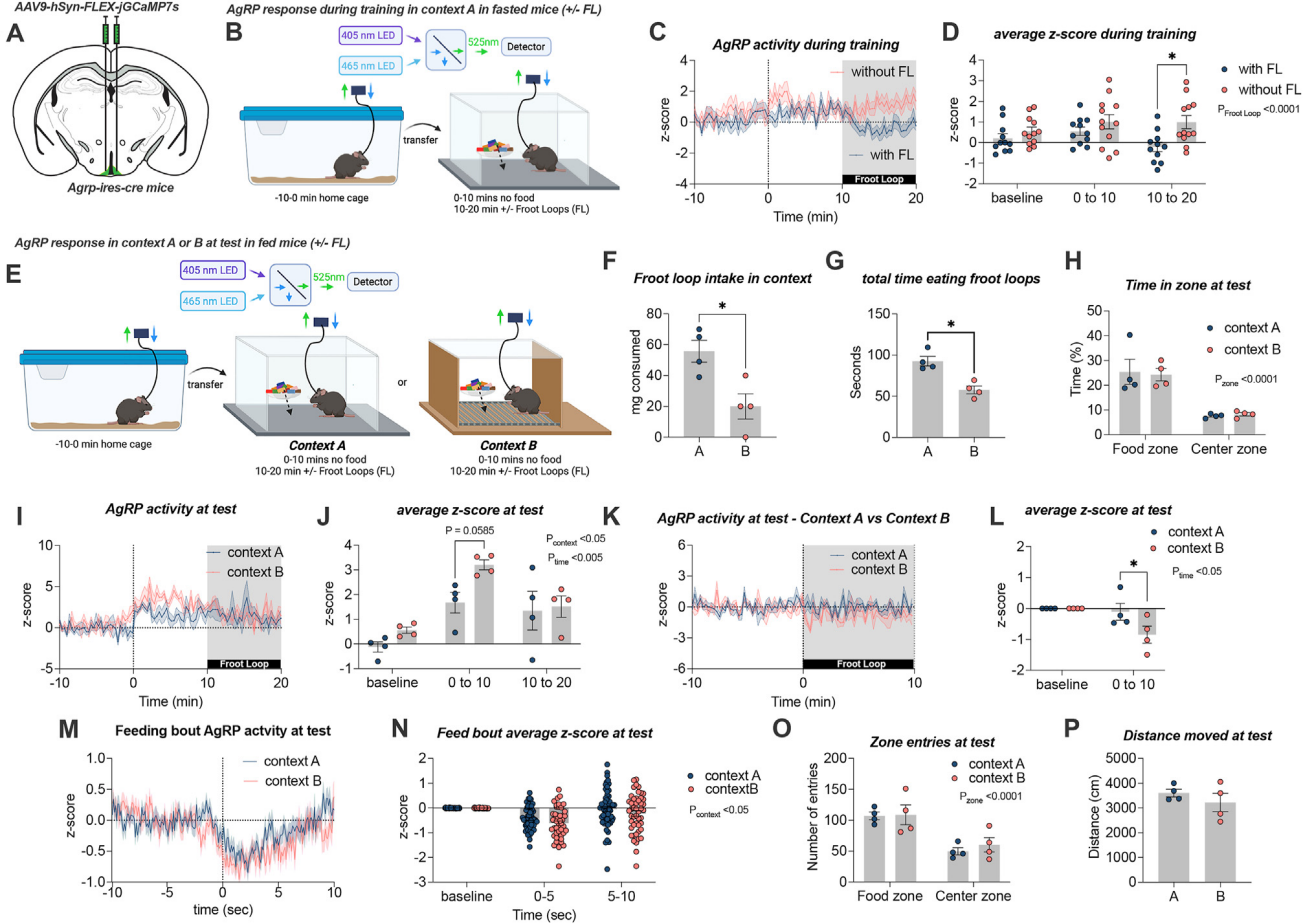
**Context-specific AgRP neuronal population activity during CIF.** (A) *Agrp*-ires-cre mice received bilateral injections of AAV9-syn-FLEX-jGCaMP7f into the arcuate nucleus for photometry experiments. (B) Experimental overview of GCaMP7f recordings during context A training created with BioRender.com. All mice were trained under fasted conditions and received Froot Loops (FL) after 10 min. (C) The presentation of Froot Loops (FL) within context A after 10 min reduced AgRP neuronal activity (data normalised to −10 to 0 baseline period; with FL n = 11; without FL n = 12). (D) Average AgRP z-score response during training in context A at baseline (−10 to 0), 0–10 and 10–20 min after Froot Loop presentation (FL). (E) Experimental overview of AgRP GCaMP7f recordings during CIF test in *ad libitum* fed mice; created with BioRender.com. (F) Mice that underwent the CIF protocol coupled with photometry showed increased Froot Loops consumption and (G) total time eating in context A compared to context B at test (paired t-test, n = 4). (H) At CIF test, mice spent more time in the food zone compared to the centre zone (two-way RM ANOVA; main effect of zone), however no differences were observed between context A and B. (I) AgRP neuronal activity in context A and B for the entire test period; Froot Loops were presented after 10 min (data normalised to −10 to 0 baseline period; n = 4). (J) Average AgRP z-score response at baseline (−10 to 0), 0–10 and 10–20 min in context A and B at test (two-way RM ANOVA main effect of context and time, Sidak's multiple comparisons post hoc analysis; n = 4). (K) AgRP neuronal response to the presentation of Froot Loops into the test context. Time 0 represents the addition of Froot Loops into the test context (data normalised to −10 to 0 baseline period; n = 4). (L) Average AgRP z-score response prior to introducing Froot Loops (−10 to 0) and 0 to 10 in context A and B at test (two-way RM ANOVA main effect of time, Sidak's multiple comparisons post hoc analysis; n = 4). (M) AgRP neuronal activity during each feeding bout within context A and B at test. Time 0 represents contact with food (data normalised to −10 to 0 s baseline period; n = 4). (N) The average AgRP z-score response to each feeding bout within context A and B at baseline (−10 to 0 s), 0–5 and 5–10 s; (two-way RM ANOVA main effect of context; n = 56 and 48 events from 4 mice in context A and B respectively). (O) At CIF test, mice made more entries in the food zone compared to the centre zone (two-way RM ANOVA; main effect of zone), however no differences were observed between context A and B (n = 4). (P) There was no difference in distance moved at test in context A and B. Dotted line in C &I represents time mice were transferred in the context. Dotted line in K represents the time Froot Loops were added during the CIF test. Dotted line M presented the time of food contact for each feeding bout. Values are presented as mean ± SEM, ∗p < 0.05. All specific statistical information is reported in Supplemental Table 1.

To examine how the process of CIF affected AgRP neuronal activity, we monitored AgRP neuronal activity with GCaMP7f *in vivo* photometry during training (3× days; Figure 2B) and at test in context A and context B (Figure 2E). During training, the addition of Froot Loops to fasted mice was essential to significantly decrease AgRP activity (Figure 2C–D). Without the addition of Froot Loops, AgRP activity remained unchanged during context training (Figure 2C–D). After 3 days of context training in fasted mice, we tested for a CIF response in fed mice in both context A (trained context) and context B while monitoring AgRP neuronal activity (Figure 2E). First, we demonstrated that mice ate more Froot Loops and spent more time eating in the trained context A compared to context B (Figure 2F, H), showing a successful CIF response. Second, we showed that there was no difference in anxiety-like behaviour at test. Although mice spent more time (Figure 2H) and had more entries (Figure 2O) in the food zone compared to the centre zone (two-way ANOVA, main effect of zone), this was not different between context A and B. Similarly, there were no differences in distance moved during test in context A compared to context B (Figure 2P). At test, transfer into context B caused a greater increase in AgRP activity compared to context A when normalized to a baseline period prior to context transfer (Figure 2I, J), suggesting that the change in AgRP activity reflects a degree of familiarity with the context. The addition of Froot Loops initiated a significantly greater decrease in average AgRP activity over a 10-min period in context B compared to context A (Figure 2K, L). However, the majority of time in either context during the test was not spent eating (Figure 2G), therefore we analysed the AgRP response to each feeding bout in context A and context B (Figure 2M). In response to a feeding bout, the suppression of AgRP activity was mildly, but significantly greater in context B compared to context A (Figure 2N). These data indicate 1) the suppression of fasting-induced AgRP activity to Froot Loops in context A during training drives a subsequent CIF response at test when *ad libitum* fed mice are placed back in context A; 2) the suppression of AgRP activity in response to Froot Loops at test is less in context A (trained) vs context B. Thus, fed mice at test have an AgRP neuronal response profile that favours food-seeking and attenuates satiety signalling in context A compared to context B.

### 3.3 Chemogenetic activation of AgRP neurons during training does not induce CIF

**Figure 3:**
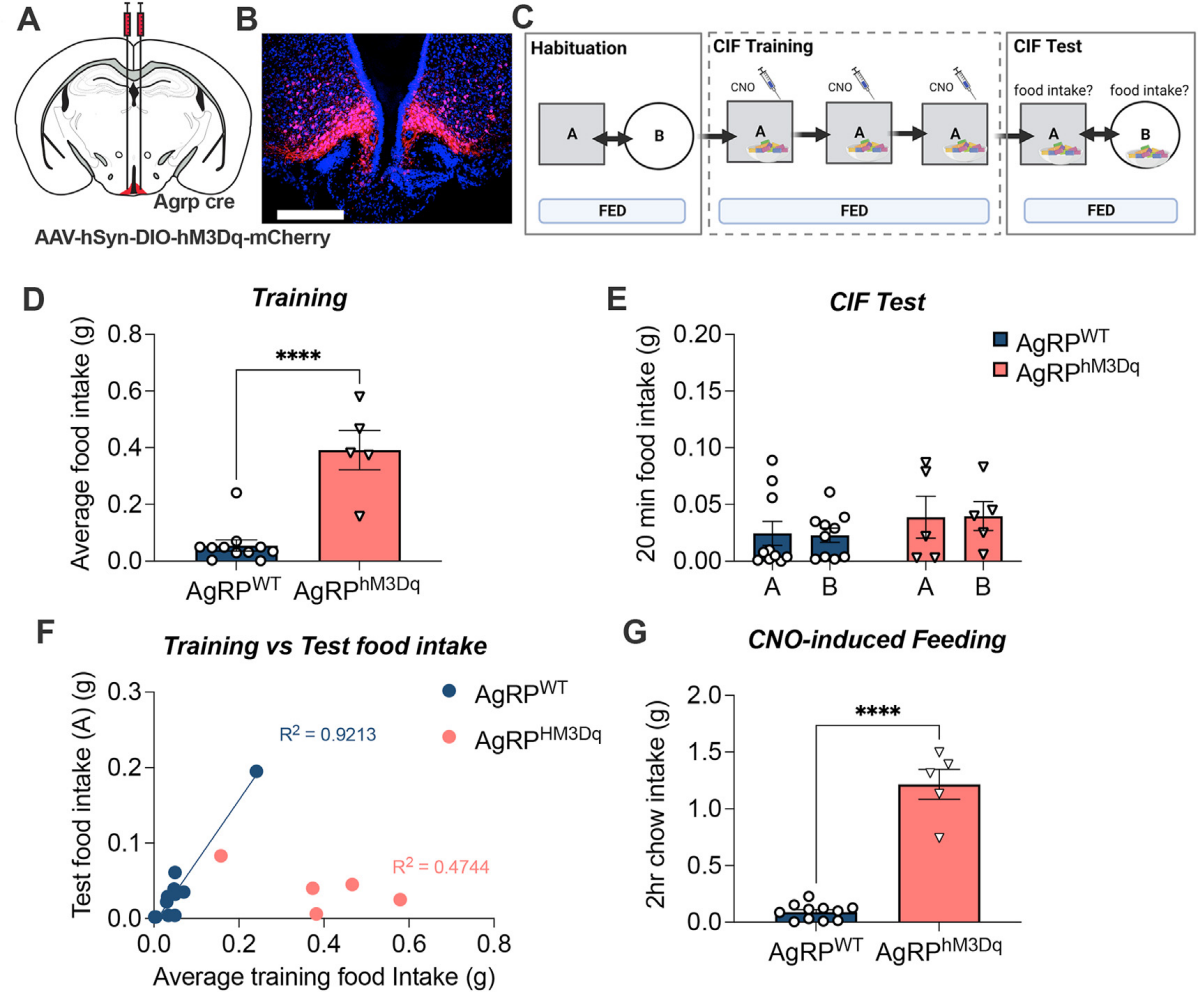
**Chemogenetic stimulation of AgRP neurons in fed mice during training.** (A) *Agrp*-ires-cre and AgRP-WT littermates received bilateral injections of AAV5-hsyn-DIO-hM3Dq-mcherry into the arcuate nucleus creating AgRP^HM3Dq^ (n = 5) and AgRP^WT^ (n = 11); respectively. (B) Representative image of AAV-hsyn-DIO-hM3Dq-mCherry in AgRP-ires-cre mice. (C) Experimental overview created with BioRender.com. All mice were trained under fed conditions and received injections of CNO (1 mg/kg) 1 h prior to training sessions. The presence of a context-induced feeding (CIF) response at test was evaluated under fed conditions by comparing intake of Froot Loops in contexts A and B. (D) Average Froot Loop intake (g) across each of the 30-min training sessions. (E) 20-min Froot Loop intake in contexts A and B under fed conditions during the context-induced feeding test. (F) Relationship between training food intake and test food intake in context A. (G) Home cage CNO-induced feeding response in AgRP^HM3Dq^ (n = 5) and AgRP^WT^ (n = 11) mice. Values are presented as mean ± SEM, student's t-test in D and G, ∗∗∗∗p < 0.0001. All specific statistical information is reported in Supplemental Table 1.

We next set out to understand whether activation of AgRP neurons during training was sufficient for fed mice to acquire CIF, in the presence of food. Firstly, *Agrp*-ires-cre mice expressing excitatory DREADDs (AgRP^hM3Dq^) (Figure 3A, B) and their wild-type littermates (AgRP^WT^) underwent CIF under fed conditions, with CNO administered daily prior to training sessions (Figure 3C). During training, AgRP^hM3Dq^ mice ate significantly greater amounts in response to CNO (average training intake p < 0.0001), indicating CNO treatment was successfully targeting AgRP neurons to drive a feeding response (Figure 3D). However, when tested for CIF in the absence of CNO, AgRP^hM3Dq^ mice did not eat more than controls, nor did they show a CIF response (Figure 3E). Moreover, these mice showed a negative relationship between acute and conditioned feeding responses (training vs test food intake in context A), in contrast to AgRP^WT^ mice whose training and test intake showed the expected positive relationship (p < 0.0001; Figure 3F). Therefore, although chemogenetic stimulation of AgRP neurons was sufficient for acute feeding within-context, this did not translate to a context-conditioned feeding response at test. The acute feeding response in fed mice was also confirmed in a home-cage environment with standard chow (Figure 3G).

### 3.4 Acute temporal optogenetic control of AgRP neurons during training drives CIF

**Figure 4:**
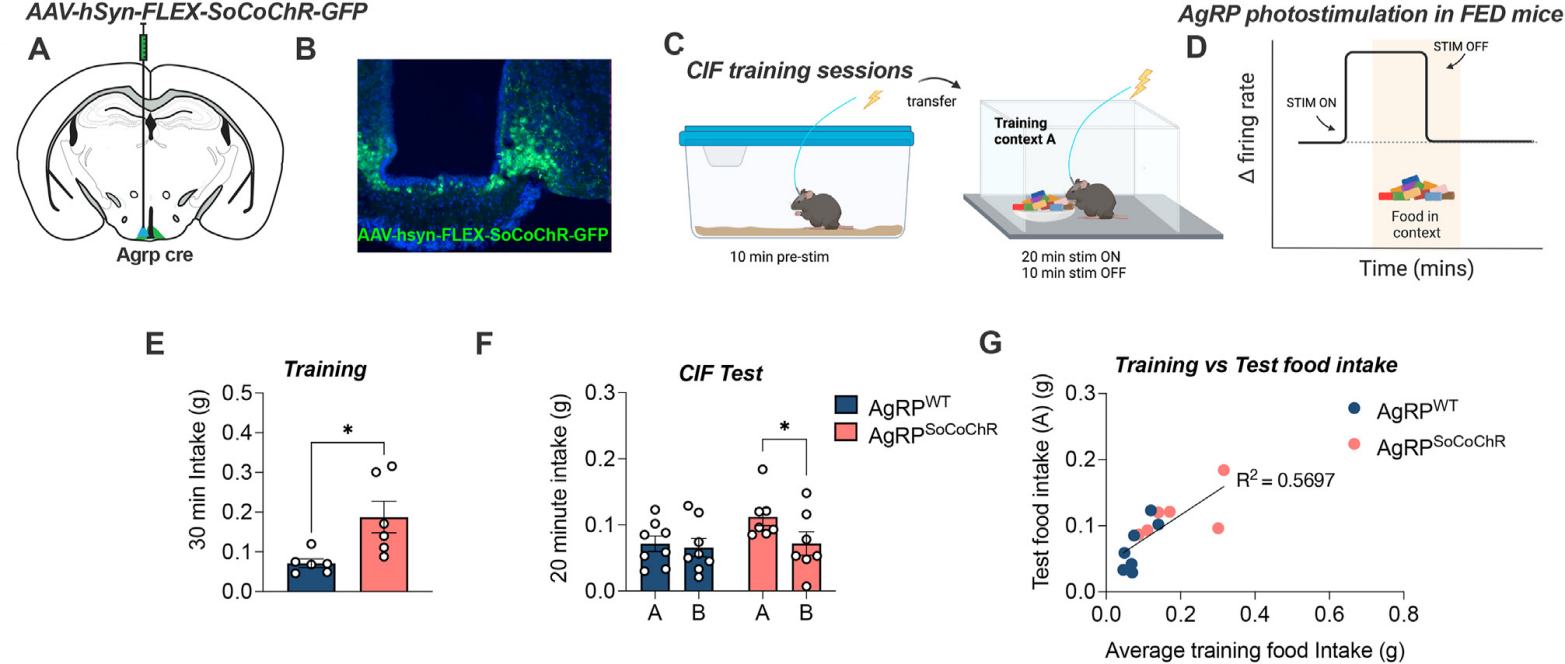
**Within-context optogenetic stimulation of AgRP neurons during training.** (A) *Agrp*-ires-cre and AgRP-WT littermates received bilateral injections of AAV9-hsyn-FLEX-soCoChR-GFP into the arcuate nucleus creating AgRP^SoCoChR^ (n = 6) and AgRP^WT^ (n = 6); respectively. An indwelling fibre optic cannula was implanted unilaterally above the injection site. (B) Representative image of GFP reporter expression in AgRP-ires-cre mice. (C) Experimental overview created with BioRender.com. Mice were habituated to contexts A and B and then trained under fed conditions. 20 Hz Photostimulation (1 s on, 3 s off) was delivered for the first 20 min of the 30-min training session, with palatable Froot Loopss available. The presence of a context-induced feeding (CIF) response at test was evaluated under fed conditions by comparing intake of Froot Loops in contexts A and B. (D) Schematic image highlighting the onset and offset of AgRP photostimulation during training relative to the time spent in context A. (E) Froot Loops intake averaged across each of the 30-min training sessions (student's t-test). (F) Context-induced feeding test; 20-min Froot Loop intake in contexts A and B under fed conditions (2-way RM ANOVA with Sidak's post hoc test for multiple comparisons). (G) Relationship between training food intake and test food intake in context A. Values are presented as mean ± SEM, ∗p < 0.05. All specific statistical information is reported in Supplemental Table 1.

Our photometry results indicated that a decrease in AgRP activity during training is necessary for a subsequent CIF response to the same context in *ad libitum* fed mice. Moreover, food and food cues rapidly inhibit AgRP neurons [19,21,25,26]. Therefore, we used an optogenetic approach, which provides greater temporal control over AgRP activity, to replicate this rapid context-specific inhibitory response to food. To this end, we repeated the CIF assay in fed mice expressing AgRP-dependent soma-targeted channelrhodopsin (AgRP^SoCoChR^) or controls (AgRP^WT^). We used a short period of photostimulation for 20 min within-context (ON–OFF within context) for each 30-min training session (Figure 4A–D). Photostimulation significantly elevated feeding in AgRP^SoCoChR^ mice within context during training (p = 0.018; Figure 4E). When assessed for CIF at the test stage without photostimulation, AgRP^SoCoChR^ mice also displayed a CIF response (p = 0.028) that was absent in AgRP^WT^ mice (A vs B; p = 0.574; Figure 4F). This suggests the short period of AgRP photostimulation, followed by within-context termination (ON–OFF in context), was sufficient to acquire CIF. Although both AgRP^hM43Dq^ mice (Figure 3) and AgRP^SoCoChR^ mice increased feeding responses during training, only the temporally-specific photostimulation and cessation within-context permitted development of a conditioned feeding response.

### 3.5 Cessation of AgRP photostimulation in the absence of food during training drives CIF

**Figure 5:**
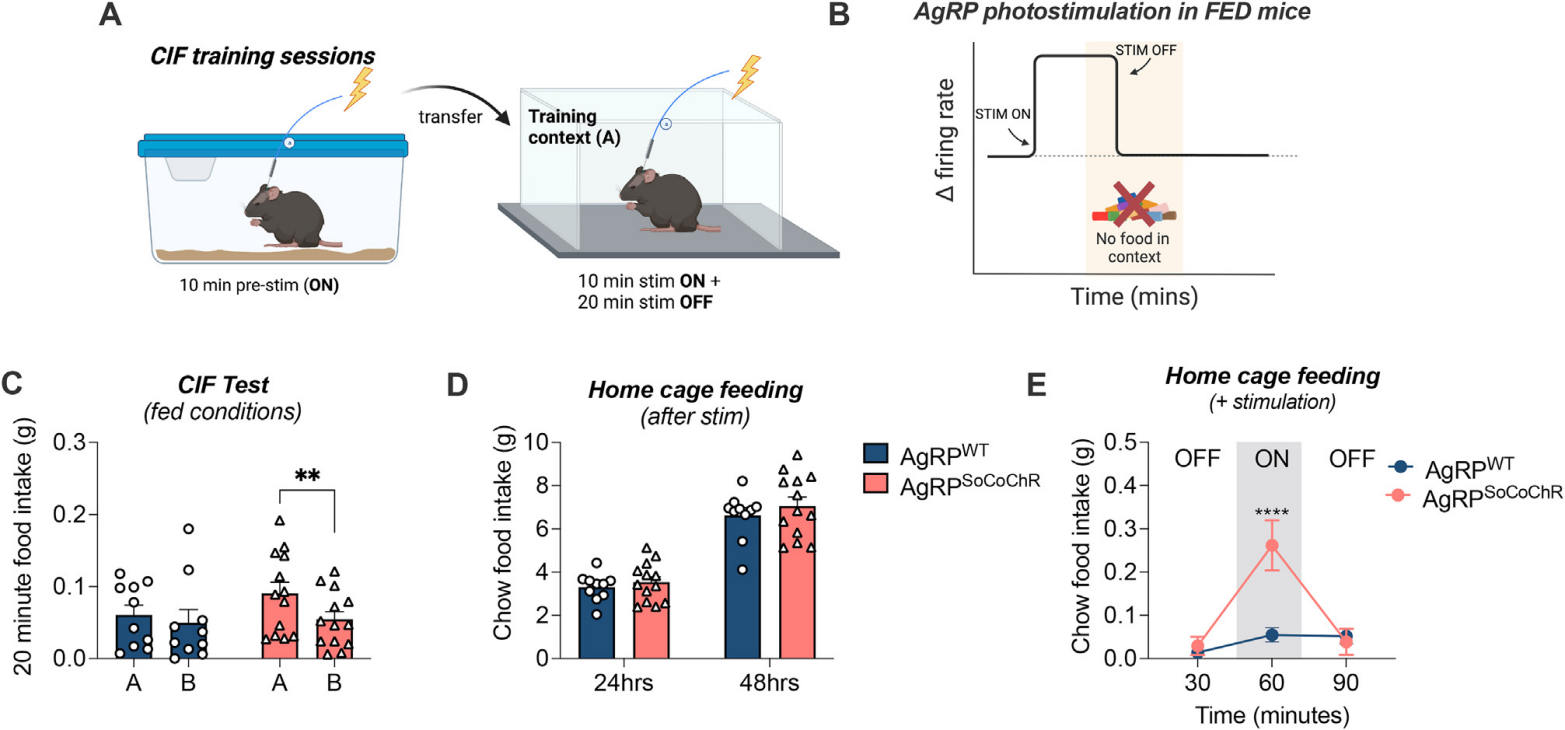
**Cessation of optogenetic stimulation of AgRP neurons in the training context.***Agrp*-ires-cre and AgRP-WT littermates received bilateral injections of AAV9-hsyn-FLEX-soCoChR-GFP into the arcuate nucleus with an indwelling fibre optic cannula implanted above the injection site, creating AgRP^WT^ (n = 10) and AgRP^SoCoChR^ (n = 13) groups, respectively. (A) Schematic created with BioRender.com outlining conditions of optogenetic stimulation given on each of the training days for the context-induced feeding experiment. Fed mice received 10 min of pre-stimulation in the home cage, followed by 10 min stimulation in the training context and an additional period without stimulation. Training was performed in the absence of food. (B) Schematic image highlighting the onset and offset of AgRP photostimulation in the absence of food during training relative to the time spent in context A. (C) Context-induced feeding Test. Froot Loop intake in 20-min testing sessions in contexts A and B. (D) Home cage feeding during the training period, measured at 24- and 48-h following the first training session. (E) Home cage chow food intake measured at 30-min intervals; prior to photostimulation (OFF), 30 min post 20 Hz stimulation (ON), and 30 min after stimulation offset (OFF). Data analysed with 2-way RM ANOVA, and Sidak's post hoc test for multiple comparisons. Values are presented as mean ± SEM, ∗∗p < 0.01, ∗∗∗∗p < 0.0001. All specific statistical information is reported in Supplemental Table 1.

The results of the previous experiment suggested the timing of AgRP neuron manipulation relative to context exposure is critical for conditioned, but not normal feeding responses in fed mice. These data support the idea that temporal inhibition of AgRP neuronal activity upon food-cue presentation and reward receipt influences learning [23,26,29]. However, whether acute silencing independent of food intake and post-ingestive feedback influences learning is unclear. In the next set of experiments, we determined whether AgRP activity dynamics alone would be sufficient for mice to acquire CIF in the absence of hunger or post-ingestive feedback from food consumption. Fed AgRP^SoCoChR^ and AgRP^WT^ mice received daily training sessions in context A with a brief period (10 min) of home-cage photostimulation that continued for the first 10 min in the training context (Figure 5A). Termination of photostimulation within context A, as witnessed by photometry during training (Figure 2C, D), mimicked the suppression of AgRP activity typically caused by the presentation food-predictive cues and food consumption (Figure 5B). Remarkably, the termination of AgRP photostimulation within context A, even in the absence of food during training, was sufficient to drive CIF in AgRP^SoCoChR^ mice, when palatable food was later offered in both contexts at test (A vs B; p = 0.005). Notably, this effect that was absent in AgRP^WT^ mice (A vs B; p = 0.619) (Figure 5C). Because no food was offered during training, we measured home cage chow intake following training sessions, but this was unaffected compared to controls at 24- or 48-h (p > 0.05; Figure 5D). Home-cage AgRP photostimulation in AgRP^SoCoChR^ mice increased chow food intake compared to AgRP^WT^ mice control mice (Figure 5E). Thus, the removal of artificially-driven AgRP activity inside the context, in the absence of fasting or food consumption, is sufficient for CIF. These data highlight that hunger-driven context-dependent learning requires an acute sustained suppression of AgRP activity.

### 3.6 Constant AgRP photostimulation during training does not induce CIF

**Figure 6:**
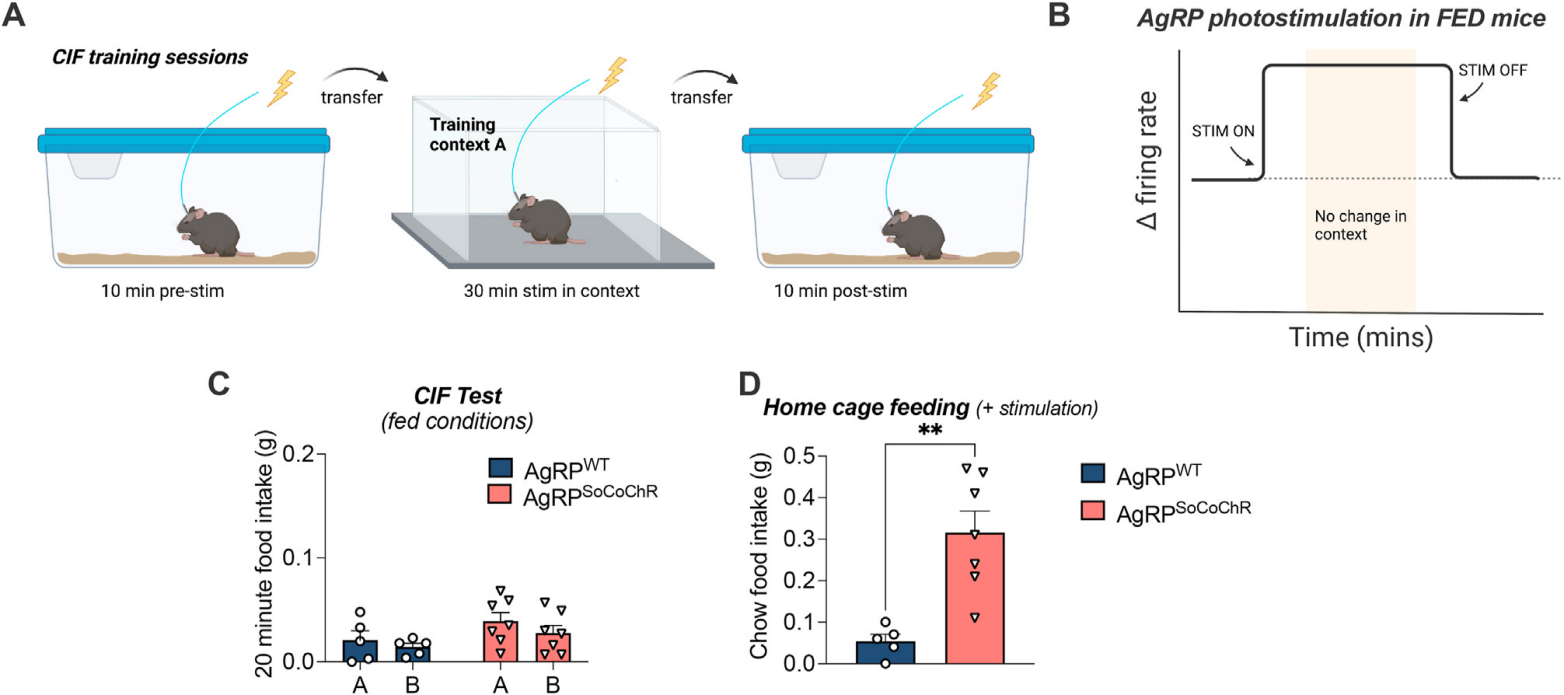
**Constant optogenetic stimulation of AgRP neurons in the training context.***Agrp*-ires-cre and AgRP-WT littermates received bilateral injections of AAV9-hsyn-FLEX-soCoChR-GFP into the arcuate nucleus with an indwelling fibre optic cannula implanted above the injection site, creating AgRP^WT^ (n = 5) and AgRP^SocoChR^ (n = 7) groups, respectively. (A) Schematic created with BioRender.com outlining conditions of optogenetic stimulation given on all training days for the context-induced feeding experiment. Fed mice received 10 min of pre-stimulation in the home cage, followed by 30 min stimulation for the entire training period in context and an additional 10-min period with stimulation once returned home cages. No food was available during training sessions. (B) Schematic image highlighting the onset and offset of AgRP photostimulation in the absence of food during training relative to the time spent in context A. (C) Context-induced feeding Test. Froot Loop intake in 20-min testing sessions in contexts A and B. (D) Home cage feeding with AgRP photostimulation (student's t-test). Values are presented as mean ± SEM, ∗∗p < 0.01. All specific statistical information is reported in Supplemental Table 1.

To reinforce the idea that the suppression of AgRP activity within the training context drives a CIF response, we next examined whether constant AgRP photostimulation during training affected the expression of a CIF response at test. For training sessions, fed AgRP^SoCoChR^ and AgRP^WT^ mice received a brief period (10 min) of home-cage photostimulation (pre-stim) that continued for the entire 30-min session within the training context (Figure 6A). No food was available during AgRP photostimulation during training sessions. After training sessions in context A, mice were placed back in their home cage and received photostimulation for a further 10 min to avoid any association between a change in AgRP activity and context A (Figure 6B). Similar to AgRP^WT^ mice at test, AgRP^SoCoChR^ mice did not show a difference in food intake in context A or context B (Figure 6C). However, standard home cage AgRP photostimulation in AgRP^SoCoChR^ mice increased chow food intake compared to AgRP^WT^ mice (p > 0.05; Figure 6D). Thus, continually high AgRP activity from photostimulation during training does not induce the expression of CIF at test. These data reinforce the idea that context-dependent suppression of AgRP activity is required for CIF.

### 3.7 AgRP photoinhibition during training in fasted mice induces CIF

**Figure 7:**
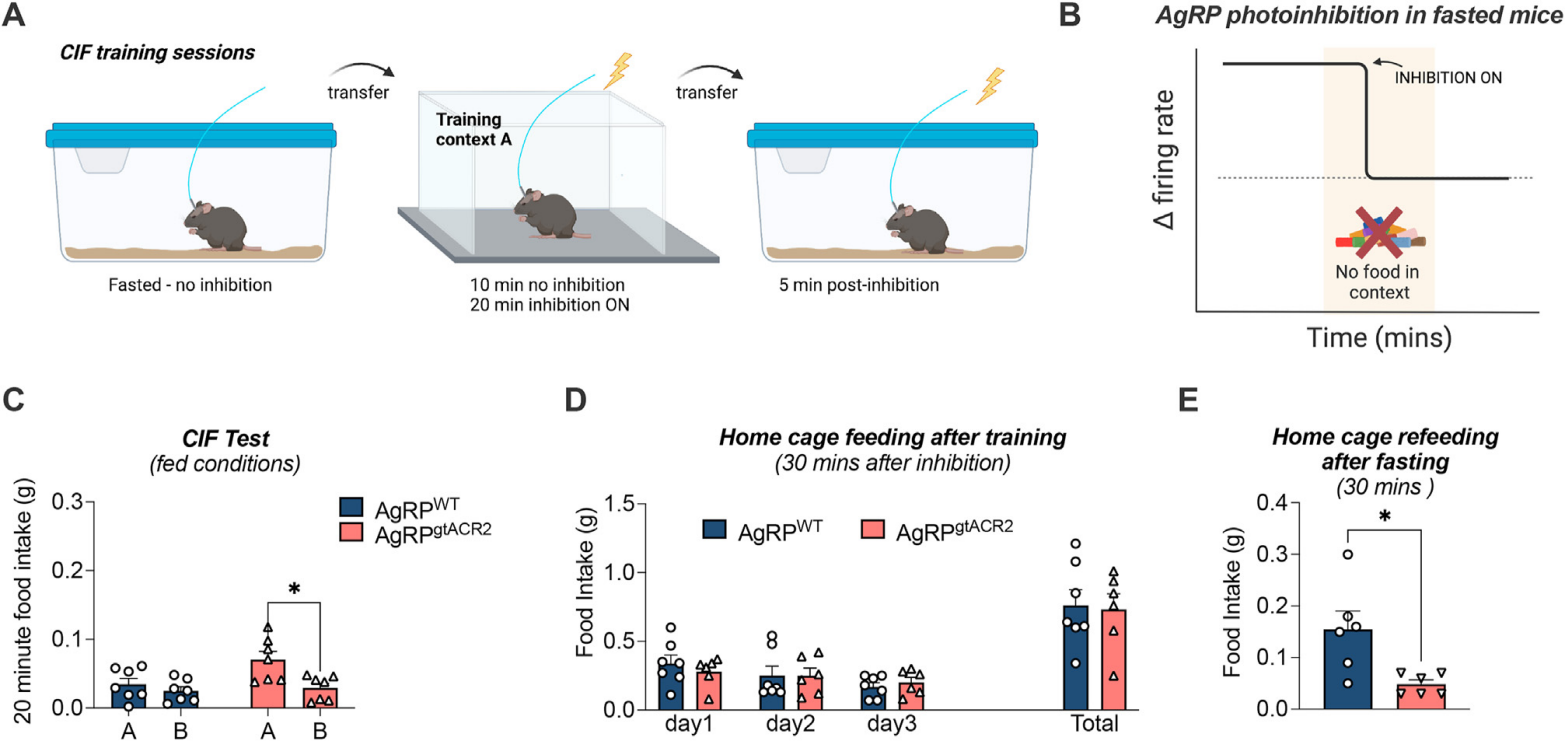
**Optogenetic inhibition of AgRP neurons in the training context.***Agrp*-ires-cre and AgRP-WT littermates received bilateral injections of AAV1-hSyn-SIO-stGtACR2-FusionRed into the arcuate nucleus with a fibre optic cannula implanted above the injection site, creating AgRP^WT^ (n = 7) and AgRP^gtACR2^ (n = 7) group. (A) Schematic created with BioRender.com outlining conditions of optogenetic inhibition in fasted mice on each of the training days. Fasted mice were placed in the training context and AgRP optogenetic inhibition was initiated after 10 min and continued for 20 min in context, as well as 5 min in the home cage. Training was performed in the absence of food. (B) Schematic image highlighting the onset of AgRP photoinhibition in fasted mice during training relative to the time spent in context A. (C) Context-induced feeding Test. Froot Loop intake in 20-min testing sessions in contexts A and B (2-way RM ANOVA, and Sidak's post hoc test for multiple comparisons). (D) Home cage refeeding after the training period, measured minutes following each training session. (E) Home cage chow food intake with AgRP photoinhibition after fasting (students t-test). Values are presented as mean ± SEM, ∗p < 0.05, ∗∗p < 0.01. All specific statistical information is reported in Supplemental Table 1.

The studies presented above used the onset and termination of AgRP photostimulation to define temporal changes in AgRP activity in fed mice, however this approach lacks a physiologically relevant stimulus to elevate AgRP activity. Therefore, in the final experiment we photoinhibited AgRP activity in context A during training sessions in fasted AgRP^gtACR2^ and AgRP^WT^ mice in the absence of food (Figure 7A, B). Photoinhibition of fasting-induced AgRP activity during training sessions induced a CIF response at test in AgRP^gtACR2^ but not AgRP^WT^ mice (Figure 7C). The cessation of photoinhibition after training sessions did not affect 30-min refeeding when mice were returned to home cages after training sessions (Figure 7D). In a separate experiment, inhibition of AgRP activity after a short 3-h fasting period significantly suppressed 30-min home-cage food intake in AgRP^gtACR2^ compared to AgRP^WT^ mice. These data indicate the temporal inhibition of fasting-induced AgRP activity within a training context, and in the absence of food, drives a subsequent CIF response at test.

## 4 Discussion

In this series of studies, we used fibre photometry to measure calcium activity in AgRP neurons, as well as optogenetic and chemogenetic manipulations of AgRP neurons to examine the reciprocal interactions between hunger and memory in a context-induced feeding (CIF) assay. We first demonstrated fasting was necessary for acquisition of CIF despite offering the same palatable food in an alternative context at test. Our results are consistent with previous studies in mice and rats showing a similar prerequisite of food restriction for learning [7,8,30]. Further, we extended these findings to show that i.p. administration of ghrelin was not sufficient to acquire CIF under our experimental conditions, despite ghrelin having known actions on AgRP neurons [19,[31], [32], [33]]. However, ghrelin treatment showed a trend for increased food intake during training and showed significant behavioural differences during CIF test, including greater eating bouts and eating time without showing a difference in total food intake at test. This lack of difference at test likely reflects insufficient training days to induce a CIF response rather than the absence of an effect of ghrelin.

Having established a working model to examine CIF, we sought to understand a neural mechanism through which fasting facilitates this context-conditioned feeding response. We hypothesised that discrepancies between fed and fasted mice could be attributed to the activity of AgRP neurons, which are more active under fasted conditions [10] and are silenced by food and food-predictive sensory cues [10,19,21,26]. Indeed, palatable food has a greater effect on AgRP neuronal silencing in fasted compared to fed mice [25]. Our photometry recordings showed that food reinforcement (Froot Loops) within context A during training caused a strong and significant inhibition of AgRP neuronal activity, consistent with the idea that AgRP inhibition in response to food cues and consumption drives learning [23,26]. Intriguingly, AgRP inhibition to Froot Loops in fed mice during CIF test was weaker in context A (trained context), despite significantly greater consumption in context A compared to context B. AgRP neurons are well known to drive food intake, food-seeking and exploration [11,14,22] and post-ingestive feedback from caloric consumption suppresses AgRP activity in a calorie-dependent manner [[26], [27], [28]]. However, our results suggest that AgRP inhibition becomes uncoupled from caloric feedback by the experience of high Froot Loop consumption within a training context. This may be related to a context-specific prediction of expected calorie availability, since the amount of food consumed during training is correlated to the amount consumed at test. Taken together, an attenuated decrease in AgRP activity at CIF test in context A likely represents 1) weaker satiety signalling and 2) prolonged food-seeking, both of which presumably underlie increased caloric consumption. Our studies highlight the novel idea that sensory information related to previous calorie consumption (training in context A) limits the satiating properties of food within the same context in current and future feeding episodes. The mechanisms responsible may involve a yet unknown relationship between predictive sensory cues and post-ingestive caloric feedback.

One notable observation was that sustained activity of AgRP neurons using hM3DGq DREADDs did not facilitate acquisition of context-induced feeding. Another study utilising a slightly modified version of conditioned feeding, showed that activation of AgRP neurons with hM3DGq DREADDs in fed mice increased the feeding response within a training context, but failed to produce a subsequent context-induced feeding response [34]. The most important feature of chemogenetic approaches is the relatively long 6-h half-life after CNO injection [35]. In comparison to normal fasted situations in which AgRP neurons are highly active and rapidly inhibited in response to food [21,25,26], chemogenetic activation of AgRP neurons in fed mice causes a prolonged activation despite food presentation and food cues. Thus, we suggest this chemogenetic approach creates a temporal mismatch between unnaturally high AgRP neuronal activity and food consumption that does not replicate physiological changes in AgRP firing properties.

An optogenetic approach, however, can more accurately reflect the firing properties of AgRP neurons *in vivo*, and in our studies photostimulation of AgRP neurons limited to 20 min (out of 30 min) in the training context was sufficient to induce CIF in fed mice, suggesting that a fall in activity (from photostimulation ON–OFF) was necessary to learn the task. Further, our results demonstrated that artificially mirroring the temporal dynamics of AgRP activity in fed mice *in the absence of food* is sufficient to acquire CIF. Moreover, the photoinhibition of AgRP neurons in fasted mice in the training context *in the absence of food* also induced a CIF response at test. Thus, we suggest a transient shift of AgRP neurons from the active to inactive state in the training context is a salient neural event that drives fasting-mediated CIF, even in the absence of post-ingestive calorie feedback. These studies demonstrate an important distinction between chemogenetic and optogenetics approaches to study the function of AgRP neurons and suggest caution should be applied when interpreting and comparing data from AgRP studies using different approaches.

The rapid silencing of AgRP neurons (seconds) reflects sensory input from afferent neural circuits and post-ingestive gut feedback is required to sustain inhibition of AgRP neurons (tens of mins). Chen and Knight proposed several possible explanations for a temporally controlled suppression of AgRP activity, including to connect current experiences with future outcomes [36]. In this way, sensory cues of a current feeding experience, associated with post-ingestive feedback from calorie consumption (reinforcement), can change how the same sensory cues affect future feeding episodes. Our data are consistent with this possibility. For example, our temporally confined optogenetic approaches suggest the timing of the ‘OFF’ signal for AgRP neurons, as would naturally occur with food presentation and post-ingestive feedback, links the change in AgRP activity to the current contextual sensory cues. This conclusion is reinforced by studies showing constant AgRP photostimulation for the entire period in the training context did not produce a CIF response at test. However, elevated food intake continues for tens of minutes after terminating AgRP photostimulation, indicating food intake is dissociated from acute AgRP firing [22]. This effect is mediated by NPY release from AgRP neurons [37], potentially suggesting that rapid suppression of GABA release from AgRP neurons may underlie the CIF response, although the exact post synaptic mechanisms remain to be determined.

Our most striking finding was that either sustained cessation of AgRP photostimulation or photoinhibition of AgRP neurons in fasted mice was sufficient to acquire a CIF response *in the absence of food*. The key reason for this may relate to acute transient vs prolonged inhibition of AgRP activity, as prolonged inhibition likely mimics post-ingestive metabolic feedback, which confirms calorie consumption and resets AgRP firing to lower rates [[26], [27], [28]]. The fact that CIF could be acquired through the cessation of AgRP activity alone supports the idea that AgRP neural activity links current and future outcomes. In this manner, the suppression of AgRP neural activity reflects a positive reinforcement signal, which aligns with the widespread use of food rewards in reinforcement learning paradigms. Moreover, the silencing of AgRP neurons might serve as a prediction-error signal that requires post-ingestive feedback for evaluation and updating. However, there are various other stimuli known to silence AgRP activity in mice, such as the cessation of exercise [38], thermal pain [39], pup reunion to dam [40] and alcohol [20]. The acute silencing signal alone may have important consequences for learning that do not require post-ingestive feedback. Thus, inhibition of AgRP neurons to non-food cues may provide additional information about an environment, although future experiments are required to investigate this further.

It is interesting to note that AgRP neurons drive learning in passive conditioning studies, such as those presented here, and using operant conditioning [14,19,21,22], suggesting they facilitate appetitive conditioning more generally. Interestingly, hunger has been used as a key motivational tool for learning in behavioural neuroscience for decades and our study uncovers an important and largely unexplored role for AgRP activity dynamics in learning and memory, as we recently discussed [29]. Moreover, the fact that activation of AgRP neurons with Gq DREADDs drives food seeking, motivation and consumption [14] but not CIF, whereas the temporal inhibition of AgRP neurons induces CIF in the absence of food intake, highlights for the first time that AgRP neurons may promote motivation and learning through distinct downstream pathways. The nature of these pathways remains to be elucidated, however elevated activity of the insular cortex, lateral hypothalamus, central amygdala and lateral septum have been demonstrated following CIF [41]. AgRP neurons influence the firing of insular cortex neurons to visual cues that predict food availability via a relay from the paraventricular thalamus and basolateral amygdala [24], suggesting activation of AgRP neurons modulates the encoding of information across various brain regions. Moreover, AgRP neurons are regulated by direct inputs from DMH GABAergic neurons co-expressing the leptin receptor (lepr) and prodynorphin (pdyn) (DMH^lepr/pdyn^), and likewise, this projection is active in response to food-predictive cues; suggesting DMH^lepr/pdyn^ neurons contribute to the rapid silencing of AgRP neurons to food cues [42]. Inhibition of these upstream pathways also increases the time taken to acquire a visual discrimination task for food rewards [23] implying that AgRP neurons influence the acquisition of this task, although a specific role for AgRP neurons was not examined in that study.

The exact mechanisms through which our CIF protocol promotes context-specific food intake remain unknown. However, the ability of food rewards to increase dopamine release and VTA dopamine neuron activity [19,43] may suggest a role for dopamine. Indeed, conditioning shifts dopamine cell firing from a reward to the cues/contexts that predict those rewards [44], a process essential for learning [45]. AgRP neuron activation also increases motivation by acting on dopaminergic pathways in the nucleus accumbens [19,20]; a site important for associative reward memory [46]. If dopamine circuits play a role in a CIF response, our results suggest the intriguing possibility that AgRP inhibition, rather than activation, is a critical event that facilitates context-specific dopamine release. Indeed, a recent study provides evidence for this idea whereby chemogenetic activation of AgRP neurons reduced the number of active VTA dopamine neurons following intragastric Ensure infusion [47]. Moreover, intragastric infusion of Ensure, which suppresses AgRP activity during and up to 20 min post infusion [26,27], is associated with enhanced dopamine release over similar time frames [47]. Thus, the inhibition of AgRP activity may be an important neural event promoting cue/context dependent VTA dopamine cell firing and release in terminal regions, although future experiments are required to experimentally address this hypothesis.

In summary, our results show that AgRP neurons mediate the acquisition of CIF, and importantly, highlight that the timing of AgRP silencing relative to context exposure is likely responsible for CIF acquisition. This silencing may confer a learning signal in the absence of post-ingestive feedback, as evidenced by a conditioned response in those experiments where mice were trained in the absence of food. More broadly, our results highlight that dynamic changes in AgRP activity influence learning and memory, and pinpoint a neural population responsible for the well-established effects of hunger on associative learning. We suggest that the magnitude of AgRP suppression to a salient signal, as well as the length of that suppression, underlies the learning potential embedded within the firing properties of AgRP neurons. Furthermore, finding a way to disrupt the formation of conditioned feeding memories may be useful to help reduce overeating, food cravings and the development of obesity.

## Disclosure statement

The authors have nothing to disclose.

## Data Availability

Data will be made available on request.
