# Supplementary material for: Acute inhibition of hunger-sensing AgRP neurons promotes context-specific learning in mice
Source: Mol Metab. 2023 Sep 9;77:101803. doi: 10.1016/j.molmet.2023.101803 (PMC10523265; doi:10.1016/j.molmet.2023.101803)
Supplement: Multimedia component 1 [file mmc1.docx]

**Appendix 1. Statistical test details and results for all analyses**

| **Figure** | **Statistical test** | **Group n** | **Main analysis result** | **Significant post-hoc multiple comparisons** |
| --- | --- | --- | --- | --- |
| **1B** | 2-way RM ANOVA | Fed (n = 10)  Ghrelin (n = 10)  Fasted (n = 9) | Training day *F(*1.796, 48.50) = 6.549; **p = 0.040**  Treatment F*(*2, 27) = 17.79; **p < 0.0001**  Training day x Treatment *F*(4, 54) = 4.645; **p = 0.0027** | Dunnett’s multiple comparisons test (all comparisons to fed group):  Day 1: Fasted > Fed; **p = 0.0070**  Day 2: Fasted > Fed; **p < 0.0001**  Day 3: Fasted > Fed; **p < 0.0001**  Day 1: Ghrelin - Fed; p = 0.073  Day 2: Ghrelin - Fed; p = 0.181  Day 3: Ghrelin - Fed; p = 0.311 |
| **1C** | 2-way RM ANOVA |  | Treatment F(1, 26) = 7.127; **p = 0.0129**  Context F(2, 26) = 5.182; **p = 0.0128**  Treatment x Context F(2, 26) = 3.199; p = 0.057 | Sidak’s multiple comparison’s test:  Fasted: A > B; **p = 0.0074** |
| **1D** | One-way ANOVA |  | Treatment *F*(2, 26) = 3.199; **p = 0.0573** | Dunnett’s multiple comparisons test (all comparisons to fed group):  Fasted > Fed; **p = 0.0340** |
| **1E** | Simple linear regression (Fed Training vs Test discrimination A-B)  Simple linear regression (Ghrelin Training vs Test discrimination A-B)  Simple linear regression (Fasted Training vs Test discrimination A-B) |  | Fed *F*(1, 8) = 0.313; R^2^ = 0.037; **p = 0.591**  Ghrelin *F*(1, 8) = 2.308; R^2^ = 0.224; **p = 0.167**  Fasted *F*(1, 6) = 3.204; R^2^ = 0.348; **p = 0.095** |  |
| **1F** | 2-way ANOVA |  | Behaviour *F*(2, 78) = 464.8; **p <0.0001**  Treatment *F*(2, 78) < 0.0001; p = 0.9999  Behaviour x Treatment *F* (4, 78) = 7.452; **p <0.0001** | Sidak’s multiple comparison’s test:  Eating food: Fasted > Fed; **p = 0.0234**  Non-food behaviours: Fed > Fasted; **p = 0.0004** |
| **1G** | 2-way ANOVA |  | Behaviour *F*(3, 104) = 3144;  **p < 0.0001**  Treatment *F*(2, 104) < 0.0001; **p >0.999**  Behaviour x treatment *F* (6, 104) = 2.253; **p = 0.0439** | Sidak’s multiple comparison’s test:  Stationary: Fasted > Fed; **p = 0.0439** |
| **1H** | 2-way RM ANOVA |  | Treatment *F*(2, 26) = 0.7183; p = 0.4991  Context *F*(1, 26) = 29.17; **p < 0.0001**  Treatment x Context *F*(2, 26) = 0.7257; p = 0.4935 | Sidak’s multiple comparison’s test:  Fasted: A > B; **p = 0.0027**  Ghrelin:A > B; **p = 0.0075** |
| **1I** | 2-way RM ANOVA |  | Treatment *F*(2, 26) = 1.083; p = 0.353  Context *F*(1, 26) = 19.78; **p < 0.0001**  Treatment x Context *F*(2, 26) = 2.629; p = 0.0912 | Sidak’s multiple comparison’s test:  Fasted: A > B; **p =** **0.039**  Ghrelin:A > B; **p = 0.001** |
| **1J** | 2-way RM ANOVA |  | Treatment *F*(2, 26) = 3.03; **p = 0.057**  Context *F*(1, 26) = 14.43; **p = 0.0004**  Treatment x context *F*(2, 26) = 0.3008; p = 0.7415 | Sidak’s multiple comparison’s test:  Fasted: A > B; **p = 0.029** |
| **Figure** | **Statistical test** | **Group n** | **Main analysis result** | **Significant post-hoc multiple comparisons** |
| **2D** | 2-way RM ANOVA | With Froot Loops (n=11)  Without Froot Loops (n=12) | Froot Loop *F*(1, 21) = 4.94; p = 0.0374  Time *F*(1.87, 39.26) = 2.959; p = 0.0668  Context x Froot Loop *F*(2, 42) = 3.065 p = 0.0572 | Sidak’s multiple comparison’s test:  10-20 Context A -Context B; **p = 0.019** |
| **2F** | Paired two tailed t test | Context A (n=4)  Context B (n=4) | t(3) = 3.888; **p = 0.03** |  |
| **2G** | Paired two tailed t test |  | t(3) = 4.583; **p = 0.019** |  |
| **2H** | 2-way ANOVA |  | Context *F*(1, 12) = 0.0087; p = 0.9270  Zone *F*(1, 12) = 35.83; **p < 0.0001**  Context x Zone *F*(1, 12) = 0.1046; p = 0.7520 |  |
| **2J** | 2-way RM ANOVA |  | Context *F*(1, 18) = 5.270; **p = 0.034**  Time *F*(2, 18) = 13.75; **p = 0.0002**  Context x Time *F*(2, 18) = 1.317; p = 0.2925 | Sidak’s multiple comparison’s test:  0-10 Context A -Context B; **p = 0.059** |
| **2L** | 2-way RM ANOVA |  | Context *F*(1, 12) = 3.662; p = 0.078  Time *F*(1, 12) = 6.153; **p = 0.029**  Context x Time *F*(1, 12) = 3.662; p = 0.078 | Sidak’s multiple comparison’s test:  0-10 Context A -Context B; **p = 0.038** |
| **2N** | 2-way RM ANOVA | Context A (n=56 events from 4 mice)  Context B (n=48 events from 4 mice) | Context *F*(1, 306) = 3.919 ; **p = 0.05**  Time *F*(2, 306) = 25.55; **p < 0.0001**  Context x Time *F*(2, 306) = 0.8949; p = 0.4097 |  |
| **2O** | 2-way ANOVA | Context A (n=4)  Context B (n=4) | Context *F*(1, 12) = 0.3235; p = 0.58  Zone *F*(1, 12) = 24.11; **p < 0.0004**  Context x Zone *F*(1, 12) = 0.1651; p = 0.6917 |  |
| **Figure** | **Statistical test** | **Group n** | **Main analysis result** | **Significant post-hoc multiple comparisons** |
| **3D** | Unpaired t test  (Average training food intake) | AgRP^WT^ (n = 11)  AgRP^hM3Dq^ (n = 5) | t(14) = 6.281; **p < 0.0001** |  |
| **3E** | 2-way RM ANOVA |  | Context *F*(1, 13) = 0.0006; p = 0.9794  Genotype *F*(1, 13) = 1.648; p = 0.2216  Context x Genotype *F*(1, 13) = 0.01304; p = 0.9108 |  |
| **3F** | Simple linear regression (AgRP^WT^ average training food intake vs Test in A)  Simple linear regression (AgRP^hM3Dq^ average training food intake vs Test in A) |  | AgRP^WT^ *F*(1, 9) = 105.4; R^2^ = 0.9213; **p < 0.0001**  AgRP^hM3Dq^ *F*(1, 3) = 2.7808; R^2^ = 0.4744; p = 0.1984 |  |
| **3G** | Unpaired t test  (Average home cage chow intake) |  | t(14) = 12.36; **p<0.0001** |  |
| **Figure** | **Statistical test** | **Group n** | **Main analysis result** | **Significant post-hoc multiple comparisons** |
| **4E** | Unpaired t test  (Average training food intake) | AgRP^WT^ (n = 6-7)  AgRP^SoCoChR^ (n = 6) | t(10) = 2.820; **p = 0.0182** |  |
| **4F** | 2-way RM ANOVA |  | Context *F*(1, 13) = 6.852; **p = 0.0213**  Genotype *F*(1, 13) = 1.675; p = 0.2181  Context x Genotype *F*(1, 13) = 3.864; **p = 0.0711** | AgRP^SoCoChR^ A > B; **p = 0.0156** |
| **4G** | Simple linear regression (AgRP^WT^ and AgRP^SocoChR^ average training food intake vs Test in A). |  | *F*(1, 11) = 14.56; R^2^ = 0.5697; **p = 0.0029** |  |
| **Figure** | **Statistical test** | **Group n** | **Main analysis result** | **Significant post-hoc multiple comparisons** |
| **5C** | 2-way RM ANOVA | AgRP^WT^ (n = 10)  AgRP^SoCoChR^ (n = 13) | Context *F*(1, 21) = 8.249; **p = 0.0091**  Genotype *F*(1, 21) = 0.8132; **p = 0.3774**  Context x Genotype *F*(1, 21) = 2.421; p = 0.1346 | Sidak’s multiple comparison’s test:  AgRP^SoCoChR^ A > B; **p = 0.0059** |
| **5D** | 2-way ANOVA |  | Time *F*(1, 42) = 110.8; **p<0.0001**  Genotype *F*(1, 42) = 0.3082; p = 0.3082  Time x Genotype *F*(1, 42) = 0.1044; p = 0.7482 |  |
| **5E** | 2-way ANOVA | AgRP^WT^ (n = 8)  AgRP^SoCoChR^ (n = 6) | Time *F*(2, 36) = 14.95; **p < 0.0001**  Genotype *F*(2, 36) = 10.19; **p = 0.0029**  Time x Genotype *F*(2, 36) = 10.03; **p = 0.0003** | Sidak’s multiple comparison’s test:  AgRP^WT^ vs AgRP^SocoChR^: 60 mins; **p < 0.0001** |
| **Figure** | **Statistical test** | **Group n** | **Main analysis result** | **Significant post-hoc multiple comparisons** |
| **6C** | 2-way RM ANOVA | AgRP^WT^ (n = 5)  AgRP^SoCoChR^ (n = 7) | Context *F*(1, 10) = 3.388; p = 0.0955  Genotype *F*(1, 10) = 2.599; p = 0.1380  Context x Genotype *F*(1, 10) = 0.2536 p = 0.6255 | Sidak’s multiple comparison’s test:  AgRP^SoCoChR^ A - B; p = 0.1890 |
| **6D** | Unpaired t test  (Home cage food intake) |  | t(10) = 4.11; **p = 0.0021** |  |
| **Figure** | **Statistical test** | **Group n** | **Main analysis result** | **Significant post-hoc multiple comparisons** |
| **7C** | 2-way RM ANOVA | AgRP^WT^ (n = 7)  AgRP^gtACR2^ (n = 7) | Context *F*(1, 12) = 6.737; **p = 0.0234**  Genotype *F*(1, 12) = 7.668; **p = 0.0170**  Context x Genotype *F*(1, 12) = 2.582; p = 0.1341 | Sidak’s multiple comparison’s test:  AgRP^gtACR2^ A > B; **p = 0.0232** |
| **7E** | Unpaired t test  (Home cage food intake) | AgRP^WT^ (n = 6)  AgRP^gtACR2^ (n = 6) | t(10) = 2.957; **p = 0.0144** |  |
